# Perivascular space dysfunction in cerebral small vessel disease is related to neuroinflammation

**DOI:** 10.1093/brain/awae357

**Published:** 2024-11-07

**Authors:** Hui Hong, Daniel J Tozer, Yutong Chen, Robin B Brown, Audrey Low, Hugh S Markus

**Affiliations:** Department of Clinical Neurosciences, University of Cambridge, Cambridge CB2 0QQ, UK; Department of Radiology, Second Affiliated Hospital of Zhejiang University, School of Medicine, Hangzhou 310009, China; Department of Clinical Neurosciences, University of Cambridge, Cambridge CB2 0QQ, UK; Department of Clinical Neurosciences, University of Cambridge, Cambridge CB2 0QQ, UK; Department of Clinical Neurosciences, University of Cambridge, Cambridge CB2 0QQ, UK; Department of Clinical Neurosciences, University of Cambridge, Cambridge CB2 0QQ, UK; Department of Clinical Neurosciences, University of Cambridge, Cambridge CB2 0QQ, UK

**Keywords:** neuroinflammation, cerebral small vessel disease, perivascular space, blood–brain barrier

## Abstract

Enlarged perivascular spaces are a feature of cerebral small vessel disease, and it has been hypothesized that they might reflect impaired glymphatic drainage. The mechanisms underlying enlargement of perivascular spaces are not fully understood, but both increased inflammation and blood–brain barrier (BBB) permeability have been hypothesized to play a role. We investigated the relationship between perivascular spaces and both CNS and peripheral inflammation, in addition to BBB permeability, in cerebral small vessel disease.

Fifty-four symptomatic sporadic cerebral small vessel disease patients were studied. Perivascular spaces were quantified both using a visual rating scale and by measurement of the volume of perivascular spaces in the white matter and the basal ganglia. PET-MRI was used to measure microglial activation using the radioligand ^11^C-PK11195, and simultaneously, BBB permeability was acquired using dynamic contrast-enhanced MRI. We determined ^11^C-PK11195 binding and BBB permeability in the local vicinity of individual perivascular spaces in concentric shells surrounding the perivascular spaces. In addition, both mean ^11^C-PK11195 binding and BBB permeability in both the white matter and the basal ganglia were determined. To assess systemic inflammation, a panel of 93 blood biomarkers relating to cardiovascular disease, inflammation and endothelial activation were measured.

Within the white matter, tissue in closest proximity to perivascular spaces displayed greater ^11^C-PK11195 binding (*P* < 0.001) in the vicinity of perivascular spaces. Higher white matter perivascular spaces burden on the visual rating scale was associated with higher white matter ^11^C-PK11195 binding (ρ = 0.469, false discovery rate-corrected *P* = 0.009); values for the volume of perivascular spaces showed a similar trend. In contrast, there were no associations between the burden of basal ganglia perivascular spaces and ^11^C-PK11195 binding. No marker of perivascular spaces was correlated with blood–brain barrier permeability. There was no association between markers of perivascular spaces and blood biomarkers of systemic inflammation.

Our findings demonstrate that white matter perivascular spaces are associated with increased ^11^C-PK11195 binding, consistent with neuroinflammation playing a role in enlargement of white matter perivascular spaces. Further longitudinal and intervention studies are required to determine whether the relationship between neuroinflammation and enlarged perivascular spaces is causal.

## Teaser

Enlarged perivascular spaces, visible on MRI, are a characteristic feature of cerebral small vessel disease, but the mechanisms underlying their expansion have been unclear. Hong *et al.* provide evidence that neuroinflammation may contribute to the enlargement of perivascular spaces in white matter.

## Introduction

Cerebral small vessel disease (cSVD) causes a quarter of all strokes, is the most common pathology underlying vascular dementia^1^ and is a major contributor to the most common form of dementia in the elderly, mixed dementia.^2^ A characteristic feature of cSVD visible on MRI are enlarged perivascular spaces (PVS).^3,4^ PVS are fluid-filled compartments surrounding the small blood vessels in the brain.^5^ They form a network of spaces around brain vessels that act as a conduit for fluid transport, exchange between CSF and interstitial fluid, and clearance of waste products from the brain.^6^ It has been hypothesized that enlarged PVS might play a role in the glymphatic dysfunction that has been implicated as a disease mechanism in the pathogenesis of cSVD and, more widely, in dementia.^7^ Enlarged PVS are associated with other MRI markers of cSVD, including white matter hyperintensities (WMH), lacunes and cerebral microbleeds.^8^ It has been suggested that they might play an early role in the disease process and that deep WMH might grow around PVS, possibly reflecting the consequences of impaired interstitial fluid drainage via PVS.^9^ However, the factors contributing to PVS dysfunction in cSVD remain unclear.

It has been suggested that inflammation might play a key role in the development of PVS dysfunction.^5^ Both systemic and CNS inflammation have been reported in cSVD.^10,11^ Circulating pro-inflammatory markers have been associated with enlarged PVS, and inflammatory cells have been shown to accumulate in the PVS in *in vivo* models.^12,13^ Furthermore, enlarged PVS are seen in a variety of neuroinflammatory conditions, and it has been hypothesized that PVS play a key role in priming neuroinflammation.^14,15^ However, the precise interaction between inflammation, enlarged PVS and brain fluid dynamics still needs to be determined.^5^

Evidence for CNS inflammation in humans can be obtained by PET, using radioligands such as ^11^C-PK11195 targeted against the translocator protein (TSPO), a mitochondrial surface protein upregulated in microglial activation. Both increased global ^11^C-PK11195 binding and hot spots of increased binding have been reported in cSVD.^16,17^ However, there are few data using ^11^C-PK11195 to investigate the relationship of microglial activation to PVS. One previous study in Alzheimer’s disease found a correlation between PVS enlargement and microglial activation using ^11^C-PK11195 PET,^17^ but whether PVS dysfunction is associated with microglial activation in cSVD remains unclear. Furthermore, if CNS inflammation plays a role in PVS pathology one might expect enlarged PVS to be associated with local inflammation, but this has not been examined in humans to date. An advantage of PET ^11^C-PK11195 is that it allows not only global brain estimates of ‘neuroinflammation’ to be obtained, but also spatial information, allowing estimates within the vicinity of enlarged PVS.

Another related process that has also been implicated in PVS dysfunction is blood–brain barrier (BBB) breakdown. PVS and BBB are anatomically connected because they are both integral components of the neurovascular unit.^18^ When the BBB is compromised, fluid from the bloodstream may enter the PVS. Neuroinflammation and BBB dysfunction have been closely linked.^19^ Release of inflammatory cells can cause breakdown of the extracellular matrix and affect the integrity of the BBB. In a rodent model of white matter ischaemia, increases in hypoxia inducible factor-1α were seen, followed by infiltrating T cells and neutrophils, with matrix metalloproteinase-9 co-localizing with the inflammatory cells, followed by BBB leakage.^20^ Increased fibrinogen, and immunoglobulin deposition, have been shown in post-mortem brains from patients with cSVD,^21,22^ consistent with BBB leakage at some time, and an increased CSF-to-serum albumin ratio, a marker of increased BBB permeability, has been reported in cSVD.^23,24^ Increased BBB permeability can be measured non-invasively, using dynamic contrast-enhanced MRI (DCE-MRI) to measure the T_1_ relaxation time change after administration of a gadolinium-based contrast agent. Increased permeability has been reported in cSVD, not only in white matter lesions but also in ‘normal-appearing white matter’, and is correlated with radiological cSVD severity.^25^ However, only one study has directly assessed the association between the burden of enlarged PVS and BBB permeability in the brain parenchyma, yielding non-significant results.^26^ Therefore, the association between BBB permeability and PVS remains unclear.

The objective of this study was to explore the relationship between PVS dysfunction and inflammation, in addition to BBB permeability in cSVD. To assess PVS dysfunction, we used the PVS visual rating score, which provides information on PVS location, in addition to quantitatively measured PVS volume. We correlated these PVS measures with markers of neuroinflammation (^11^C-PK11195 PET), with circulating biomarkers of systemic inflammation and with BBB permeability measured using DCE-MRI. Using the spatial information provided by both ^11^C-PK11195 PET and DCE-MRI, we also determined whether microglial activation and BBB permeability were increased in the local vicinity of PVS.

## Materials and methods

### Participants

**Figure 1:**
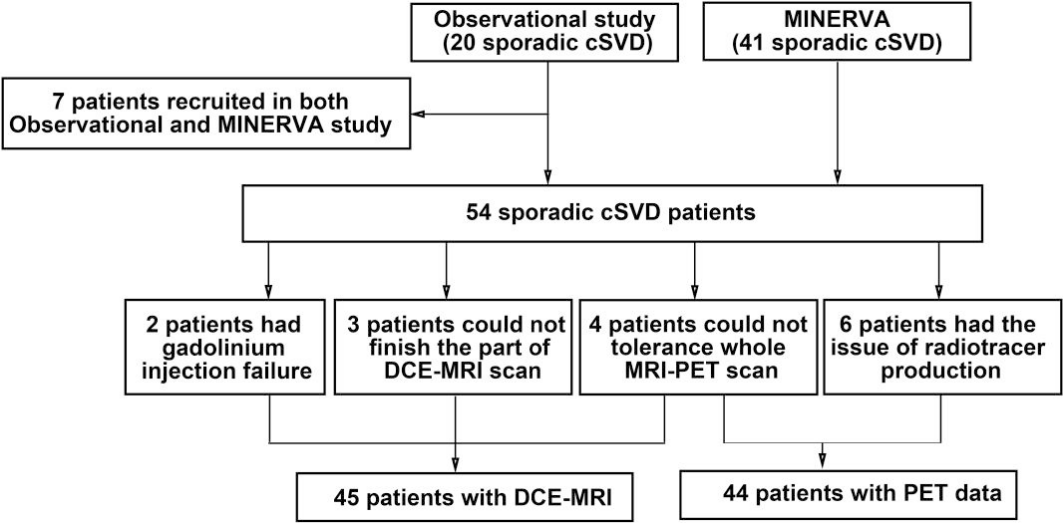
**Cohort flow chart.** cSVD = cerebral small vessel disease; MINERVA = MINocyclinE to Reduce inflammation and blood–brain barrier leakage in small vessel disease; DCE-MRI = dynamic contrast-enhanced MRI.

Symptomatic cSVD subjects were recruited prospectively as part of a PET-MRI study investigating the role of neuroinflammation and BBB permeability in cSVD. The first 20 patients were recruited as part of an initial observational study^27^ and the second 41 as part of a randomized controlled trial testing whether minocycline can reduce neuroinflammation in cSVD, the MINocyclinE to Reduce inflammation and blood–brain barrier leakage in small vessel disease (MINERVA) trial (ISRCTN: 15483452).^28^ For the patients recruited as part of the MINERVA trial, only baseline data, prior to administration of any study treatment, were analysed. Seven patients were included in both the observational study and the MINERVA trial, and for these only data from the MINERVA dataset were used. Details are shown in Fig. 1.

#### Inclusion criteria

Participants were included if they had clinical evidence of cSVD as evidenced by a lacunar stroke syndrome (e.g. pure motor stroke, pure sensory stroke, sensorimotor stroke or ataxic hemiparesis, or clumsy hand dysarthria syndrome) with a corresponding acute lacunar infarct on diffusion weighted imaging (DWI) for cases imaged (clinically) within 3 weeks of stroke, or anatomically compatible lacunar infarct on fluid attenuated inversion recovery (FLAIR) sequencing/T_1_ MRI for cases imaged later after stroke (≤1.5 cm diameter) and/or symptoms of cognitive impairment. In addition, for inclusion subjects had to have confluent white matter hyperintensities on T_2_-weighted MRI (Fazekas scale score ≥ 2).^29^

#### Exclusion criteria

Exclusion criteria were as follows: unable/unwilling to consent; recorded diagnosis of dementia for consent issues; age <18 years; subcortical infarcts of >1.5 cm maximum diameter, because many of these are striatocapsular infarcts caused by embolism; cortical stroke; any stroke cause other than cSVD, including cardioembolic source, or carotid or vertebral stenosis >50% measured on North American Symptomatic Carotid Endarterectomy Trial (NASCET) criteria^30^; probable cerebral amyloid angiopathy defined by the modified Boston criteria^31^; known or suspected monogenic form of small vessel disease; renal impairment as evidenced by an estimated glomerular filtration rate ≤ 59 ml/min in view of MR contrast administration; contraindications to taking part in MRI study, e.g. pacemaker; and women of childbearing potential, pregnant or breastfeeding.

All subjects were studied ≥3 months after the last stroke to reduce the effects of acute changes in BBB permeability and neuroinflammation changes secondary to acute infarction.

### Imaging acquisition

The neuroimaging protocol included PET and MRI co-acquired on a 3 T GE SIGNA PET-MRI scanner (GE Healthcare) at the Wolfson Brain Imaging Centre in Cambridge, UK.

PET data acquisition was carried out 75 min after the injection of ^11^C-PK11195 (target injection activity 500 MBq) produced at the Wolfson Brain Imaging Centre Radiopharmaceutical Unit. The median injected ^11^C-PK11195 activity was 440 MBq (interquartile range, 401–483 MBq), with corresponding injected PK11195 mass values of 3.9 μg (interquartile range, 2.8–6.4 μg).

Simultaneous whole-brain non-contrast MRI was carried out using a 32-channel head coil (Nova Medical), including: T_1_ image acquired using axial three-dimensional fast-spoiled gradient echo sequence (BRAVO), flip angle = 12°, inversion time = 450 ms, field of view = 28 mm, slice thickness = 1 mm, number of slices = 192, reconstructed matrix size = 512 × 512; T_2_-weighted images acquired using axial T_2_ fast-spoiled gradient echo sequence angled anterior commissure–posterior commissure, flip angle = 111°, echo time (TE) = 85 ms, repetition time (TR) = 6000 ms, field of view = 22 mm, slice thickness = 5 mm, number of slices = 31, reconstructed matrix size = 1024 × 1024; T_2_ FLAIR sequence with angled anterior commissure–posterior commissure, flip angle = 160°, TR = 8800 ms, TE = 120 ms, inversion time (TI) = 2445 ms, field of view = 22 mm, slice thickness = 5 mm, number of slices = 28, reconstructed matrix size = 256 × 256; axial susceptibility-weighted imaging, with flip angle = 17°, repetition time = 40.6 ms, echo time = 24.2 ms, field of view = 22 mm, slice thickness = 2 mm, number of slices = 70, reconstructed matrix size = 256 × 256; axial diffusion tensor imaging, with angled anterior commissure–posterior commissure, with the diffusion gradients applied in 63 directions with a b-value = 1000 s/mm^2^, TE = minimum, TR = 15 763 ms, field of view = 19.2 mm, slice thickness = 2 mm, number of slices = 65–70 depending on slice angulation, reconstructed matrix size = 256 × 256; and DCE-MRI acquired with three-dimensional radiofrequency spoiled gradient echo, TR = 6.3 ms, TE = 1.784 ms, number of slices = 16, reconstructed matrix size = 256 × 256, final resolution = 0.94 mm × 0.94 mm × 3 mm, flip angles = 2°, 5°, 12°, 17°, 22° and 27°, temporal resolution = 15 s per flip angle with interphase interval 15 s (eight cycles). Gadoterate meglumine, a gadolinium-based contrast agent (Dotarem^®^) was injected at a dose of 0.025 mmol/kg.

### Imaging analysis

#### Cerebral small vessel disease imaging markers

WMH were marked on FLAIR images using Jim analysis software version 7.0.5 (Xinapse Systems Limited; http://www.xinapse.com/j-im-7-software/). Lacunes were identified by a neurologist expert rater on FLAIR images, with additional inspection of T_1_ and T_2_ sequences to exclude other lesions that can be difficult to discriminate on FLAIR alone, such as PVS. Microbleeds were identified on susceptibility-weighted imaging by a neurologist expert rater according to the BOMBS criteria.^32^

#### White matter masks

**Figure 2:**
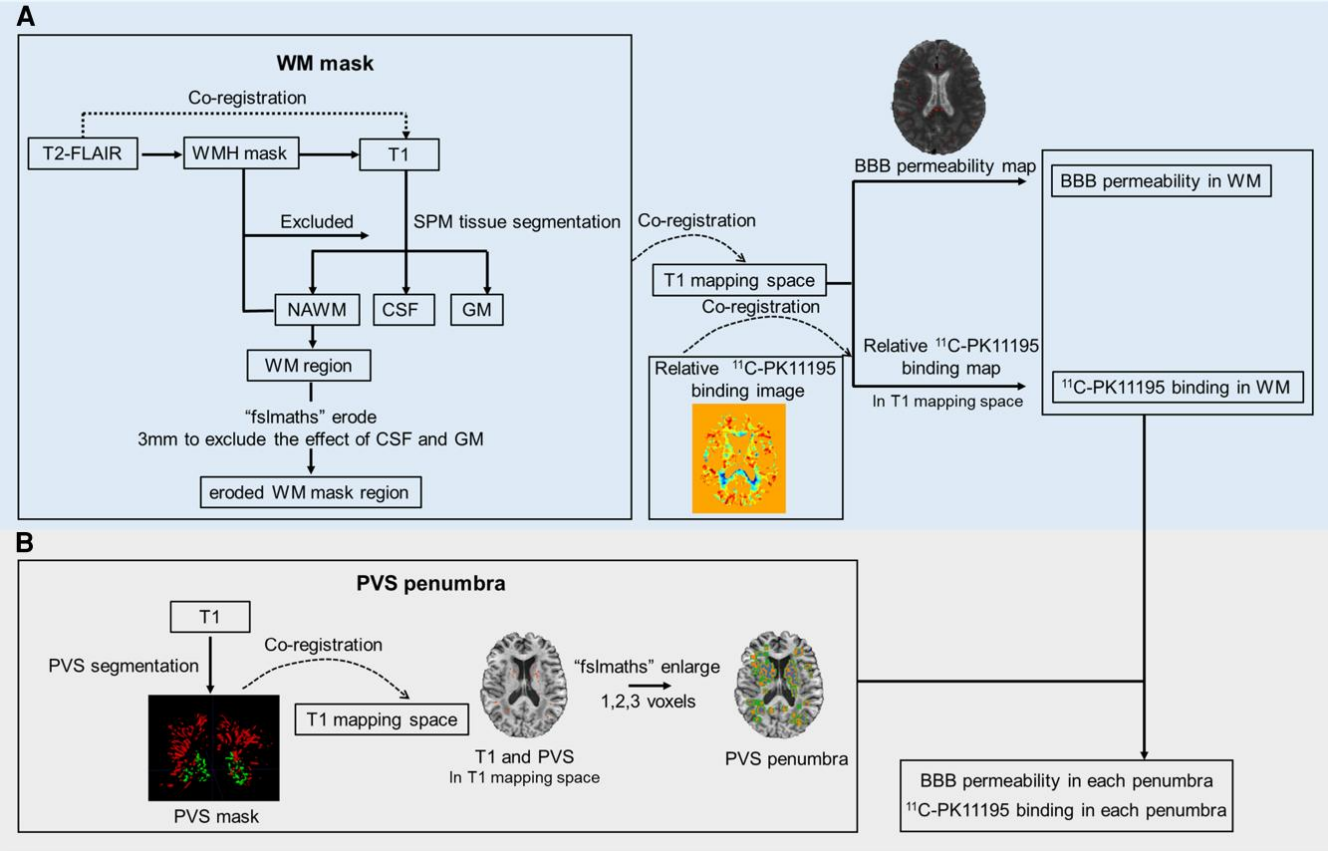
**The flowchart for the measurement of regions of interest in blood–brain barrier permeability and ^11^C-PK11195 binding.** (**A**) Measurement of blood–brain barrier (BBB) permeability and ^11^C-PK11195 binding in white matter region. (**B**) Measurement of BBB permeability and ^11^C-PK11195 binding in each PVS penumbra. GM = grey matter; PVS = perivascular space; WM = white matter.

The FLAIR image was registered to the T_1_ image using a rigid body transformation in Advanced Normalization Tools (ANTs; http://stnava.github.io/ANTs/). The resulting transformation was used to resample the WMH mask from the FLAIR image to the T_1_ image using nearest-neighbour interpolation. Each T_1_ image was processed using the ‘segment’ routine in SPM12 (https://www.fil.ion.ucl.ac.uk/spm/software/spm12/). SPM segmentation provides tissue probability maps, and volumes for each tissue class were calculated as the sum of voxels that have a probability of >0.5 of belonging to that class, after removal of voxels in the WMH mask. White matter masks were acquired by summing of white matter tissue segmented by SPM and WMH masks in T_1_ space. The masks were then eroded by 3 mm using fslmaths (https://fsl.fmrib.ox.ac.uk/fsl/fslwiki), to eliminate ventricular or grey matter contamination effectively. As shown in Fig. 2A, the white matter mask was then registered to T_1_ mapping space to acquire the BBB permeability and ^11^C-PK11195 binding in white matter. Therefore, the white matter mask was matched to the limited field of view acquired for the BBB imaging and also applied to the PET imaging.

#### Visual rating of PVS and masks

Visual rating of PVS was conducted by a trained rater based on T_2_-weighted images, cross-checking against FLAIR and T_1_-weighted images to avoid rating lacunes or WMH as PVS, following the method of Ballerini *et al*.^33^ This rating identifies the closest category on the scale of 0 (no PVS), 1 (mild; 1–10 PVS), 2 (moderate; 11–20 PVS), 3 (frequent; 21–40 PVS) or 4 (severe; > 40 PVS), based on an estimate of the number of PVS seen in the slice considered to have the highest PVS burden. PVS were rated separately in the basal ganglia and centrum semiovale.

N4 bias correction was performed on T_1_ image using ANTs, followed by skullstripping using HD-BET and rigid registration to the Montreal Neurological Institute (MNI) space. The voxel intensities within the brain region were *z*-transformed, and values below minus two and above two were clipped. PVS segmentation was performed on the processed T_1_ images using a pretrained deep learning model (https://github.com/pboutinaud/SHIVA_PVS) to produce binary lesion masks of PVS, which were visually inspected and manually corrected by a single trained rater. These were cross-checked on T_2_ and FLAIR images (registered to T_1_ space) to exclude lacunes or WMH, which can be difficult to discriminate on T_1_. To extract regional PVS volumes from the PVS masks, FreeSurfer was used to parcellate the skullstripped T_1_ images to derive regional masks of cerebral white matter (FreeSurfer labels 2 and 46) and basal ganglia (FreeSurfer labels 11, 12, 13, 50, 51 and 52); these labels were used to calculate white matter PVS (WM PVS) volume and basal ganglia (BG) PVS volume, respectively. The intra-rater reliability in PVS labelling was found to be excellent, with an intraclass correlation coefficient of 0.951 for whole-brain PVS volume, 0.914 for BG PVS volume and 0.948 for WM PVS volume assessed from an independent training set of 10 scans marked with a gap of 3 months between attempts. The correlations between PVS visual rating score and WM PVS volume and BG PVS volume were (*ρ* = 0.751) and (*ρ* = 0.703), respectively.

#### Dynamic contrast-enhanced MRI analysis

The T_1_ maps from the DCE-MRI were calculated using the standard radiofrequency spoiled-gradient echo signal equation and used to estimate the gadolinium concentration in tissue using a Patlak graphical analysis to determine influx rate (*K*_i_) as the measure of BBB permeability.^34^ Given that there is no artery in the field of view, the superior sagittal sinus was used as an arterial input function, corrected by the factor (1 − haematocrit), which is assumed to be representative of the arterial concentration of contrast agent (Supplementary Fig. 2). Global values of BBB permeability in the white matter were calculated as the mean *K*_i_ across the voxels in that region (Fig. 2A).

To validate the MRI measure of BBB permeability in a subgroup of 12 participants from the same cohort who consented to additional lumbar puncture at baseline, we previously measured CSF and serum albumin to determine the CSF-to-serum albumin ratio, an established marker of BBB permeability. The CSF-to-serum albumin ratio was 5.39 ± 1.71 mg/g and was highly correlated with the overall mean transfer coefficient (Pearson’s *r* = 0.599, *P* = 0.040).^35^

#### PET analysis

List-mode PET data were histogrammed into 55 time frames, then reconstructed into images (128 × 128 × 89 matrix; 2.0 mm × 2.0 mm × 2.8 mm voxel size) using time-of-flight ordered subsets expectation maximization, with 16 subsets, six iterations and no smoothing. Attenuation correction included the use of a multisubject atlas method and improvements to the MRI brain coil component. Image reconstruction also included corrections for random coincidences, dead time, normalization, scattered coincidences, radioactive decay and sensitivity.

SPM12 (https://www.fil.ion.ucl.ac.uk/spm/software/spm12/) was used to realign each dynamic image series. A mean realigned PET image was then used to co-register each realigned dynamic PET image series to the T_1_ BRAVO magnetic resonance image from the same scan. To estimate specific binding of ^11^C-PK11195 (BP_ND_), binding potential relative to a non-displaceable compartment was determined with a version of the simplified reference tissue model using a basis function approach, also incorporating correction for vascular binding. TSPO is expressed by cell types other than microglia, including endothelial cells. To account for this, correction of the PET signal for binding to vascular endothelial cells has been developed, and this was applied in the reference tissue kinetic model used in this work to increase the specificity for TSPO expressed in parenchymal brain tissue.^36^ This correction involves selecting 10 voxels that showed the greatest activity in the first five frames and using these to measure the vascular activity throughout the time course; this is then added to the model for determining binding potential. A control population from a separate study using the same scanner, acquisition and processing protocol was used to determine white matter tissue reference signal curves, which were then used in place of arterial plasma curves in the model fitting. A 4 mm full-width at half-maximum Gaussian was applied to the dynamic images prior to production of BP_ND_ maps using a simplified reference tissue model (Supplementary Fig. 2). Mean BP_ND_ in white matter region were calculated (Fig. 2A).

#### PVS ‘penumbra’

To evaluate the spatial correlation between PVS and BBB permeability and between PVS and ^11^C-PK11195 binding, a ‘penumbra’ around the PVS was delineated. This was defined by taking the PVS mask in T_1_ mapping space and dilating this by one, two and three voxels in all directions (Fig. 2A). These masks were further masked using white matter mask to leave only in white matter region. Mean ^11^C-PK11195 binding and BBB permeability values were obtained in each layer of the penumbra (Fig. 2B).

#### Blood biomarkers

Blood was sampled from each participant at the same time as the PET/MRI scan, centrifuged, and the serum was separated and stored at −70°C. A panel of 93 blood biomarkers relating to cardiovascular disease, inflammation and endothelial activation were measured in each participant. These comprised 92 markers from the Olink Proteomics Cardiovascular Disease III panel (https://www.olink.com/products/cvd-iii-panel/), together with measurement of C-reactive protein measured using the Siemens Dimension high-sensitivity C-reactive protein assay at the Core Biochemical Assay Laboratory, Addenbrooke’s Hospital, Cambridge, UK. To account for batch effects, the proteomic data were normalized using the plate median for each assay as the normalization factor.

### Statistical analysis

All the statistical analyses were performed in R-studio (R version 4.1.3). For demographics, the Shapiro–Wilk test was performed to assess normality for continuous variables. If the variable was normally distributed, the mean and standard deviation (SD) was used to summarize results. For variables not normally distributed, the median and interquartile range were reported. PVS volume was normalized by brain volume (white matter + grey matter) and log-transformed. To meet the requirement of data normal distribution for some statistical analysis, cube root transformation was performed for both ^11^C-PK11195 binding and BBB permeability measurements.

#### The spatial association between PVS and ^11^C-PK11195 binding and between PVS and BBB permeability

Mean BP_ND_ and mean *K*_i_ were compared across penumbra layers using ANOVA tests. *Post hoc* pairwise comparisons using paired *t*-tests were conducted to compare the difference between each pair of penumbra layers, with Bonferroni correction applied to account for multiple comparisons.

#### PVS associations with ^11^C-PK11195 binding and BBB permeability

Spearman correlation analysis was used to analyse the association between PVS visual rating score in each region (WM PVS and BG PVS) and mean BP_ND_. Linear regression analysis was used to analyse the association of mean BP_ND_ with PVS volume (whole-brain PVS volume, WM PVS volume and BG PVS volume). The volumes of whole-brain PVS, BG PVS and WM PVS were individually set as dependent variables. Mean BP_ND_ in white matter region was considered as an independent variable. Age and sex were included as covariates in all analyses. False discovery rate (FDR) correction was used to account for multiple comparisons.

For the association between mean *K*_i_ and PVS markers, Spearman correlation analysis was used to analyse the association between PVS visual rating score in each region (WM PVS and BG PVS) and mean *K*_i_. For the association of *K*_i_ with PVS volume (whole-brain PVS volume, WM PVS volume and BG PVS volume), the similar linear regression models mentioned above were conducted with *K*_i_ mean value as an independent variable instead. FDR correction was used to account for multiple comparisons.

#### The association between blood biomarkers and PVS markers

To analyse the association between PVS markers and blood biomarkers, the Shapiro–Wilk test was used to investigate the normal distribution of blood biomarkers. If it was not normally distributed, we used log or cube root transformation to approximate a normal distribution. Then Pearson correlation analysis was performed to investigate the association of PVS volumes with 93 blood biomarkers. Spearman correlation analysis was performed for the relationship between PVS visual rating score and 93 blood biomarkers. All analysis were controlled for age and sex and were adjusted for multiple comparisons using FDR.

Principal component (PC) analysis was also conducted to summarize the variation of 93 biomarkers, because the first three dimensions explained most of the variance (Supplementary Fig. 1). Pearson correlation analysis for PVS volume and Spearman correlation analysis for PVS visual rating score were used to investigate the association between PVS markers and each PC; all analyses were controlled for age and sex. The *P*-values were adjusted for multiple comparisons using the FDR procedure.

#### Associations of PVS with BP_ND_, *K*_i_ and blood biomarkers within the same model

Additional multiple linear regression analyses were conducted to investigate the association between PVS markers and ^11^C-PK11195 binding, BBB permeability and PCs of blood biomarkers within the same model. For continuous variables, the ‘lm’ function was used, with whole PVS volume, WM PVS volume and BG PVS volume as dependent variables separately. For the ordinal dependent variables, the ‘clm’ function was used instead, with WM and BG PVS visual rating score as dependent variables, respectively. The independent variables in the models were mean ^11^C-PK11195 binding, mean BBB permeability and the PCs from PC analysis of blood biomarkers, with age and sex as covariates.

## Results

### Demographics

Fifty-four cSVD subjects were included. Four subjects could not tolerate the PET-MRI scan, and in six cases the radiotracer did not pass quality control, leaving 44 for the PET analyses. Four could not tolerate the PET-MRI scans, three could not finish the DCE part of the MRI scan during the PET-MRI scan, and in two there was gadolinium injection failure, leaving 45 in the BBB analysis. The detailed cohort flow chart is depicted in Fig. 1. The distribution of demographics, cSVD imaging markers and PVS markers is shown in Table 1.

**Table 1:** Demographics, cerebral small vessel disease imaging markers and perivascular space markers

Parameter	Sporadic cSVD (*n* = 54)
Age, years	70.81 ± 10.61
Male, *n* (%)	31 (57.4%)
Education, years	12 [12–14]
**Vascular risk factors**	
Hypertension, *n* (%)	47 (87.0%)
Hypercholesterolaemia, *n* (%)	39 (72.2%)
Diabetes, *n* (%)	10 (18.5%)
Smoking, *n* (%)	31 (57.4%)
History of stroke, *n* (%)	54 (100%)
**cSVD imaging markers**	
WMH, cm^3^	20.92 [12.26–39.49]
Lacune, *n* (%)	50 (92.59%)
Microbleed, *n* (%)	26 (48.15%)
**PVS markers**	
WM PVS visual score	2.00 [2.00–3.00]
BG PVS visual score	2.00 [1.25–3.00]
Whole PVS volume, cm^3^	1.60 [1.03–2.31]
WM PVS volume, cm^3^	1.06 [0.66–1.79]
BG PVS volume, cm^3^	0.33 [0.21–0.58]

Values are presented as the mean (SD) or median [interquartile range] or *n* (%). Hypertension was defined as either systolic blood pressure of >140 mmHg, diastolic blood pressure blood pressure >90 mmHg or on treatment. Hypercholesterolaemia was defined as a random total cholesterol of >5.2 mmol/L or on treatment. Diabetes mellitus was defined as being on drug or insulin treatment. Smoking included both ex-smoker and current smoker. BG = basal ganglia; cSVD = cerebral small vessel disease; PVS = perivascular space; WM = white matter; WMH = white matter hyperintensity.

### Spatial analysis of BP_ND_ and *K*_i_ in the vicinity of PVS

**Figure 3:**
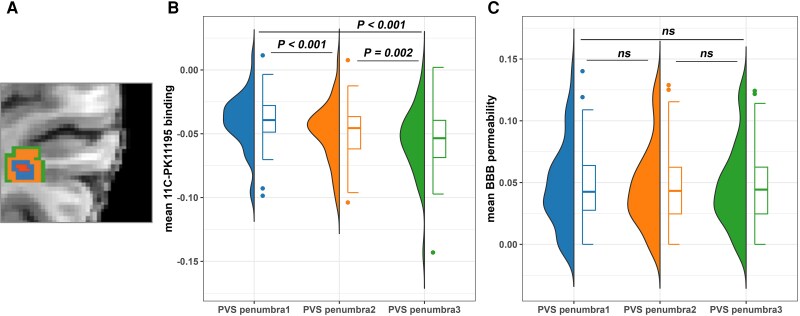
**Spatial analysis of ^11^C-PK11195 binding and blood–brain barrier permeability in the vicinity of the perivascular space.** (**A**) ‘Penumbras’ of the perivascular space (PVS): the innermost labelled region (red), enlarged PVS; the second innermost labelled region (blue), penumbra layer 1; the third innermost labelled region (orange), penumbra layer 2; the outermost labelled region (green), penumbra layer 3. (**B**) Comparison of mean ^11^C-PK11195 binding among PVS ‘penumbras’. (**C**) Comparison of mean blood–brain barrier (BBB) permeability among PVS ‘penumbras’. *ns* = not significant.

CNS inflammation, as measured by increased ^11^C-PK11195 binding, was increased in the vicinity of PVS, as indicated by a significant inverse correlation between the distance from PVS and the mean white matter BP_ND_ (*P* < 0.001); as the distance from PVS increased, there was a decrease in mean white matter BP_ND_ (Fig. 3B). In contrast, for BBB permeability there was no difference in mean *K*_i_ value (*P* = 0.338) among PVS penumbra layers (Fig. 3C), demonstrating that PVS were not associated with locally increased BBB permeability.

**Figure 4:**
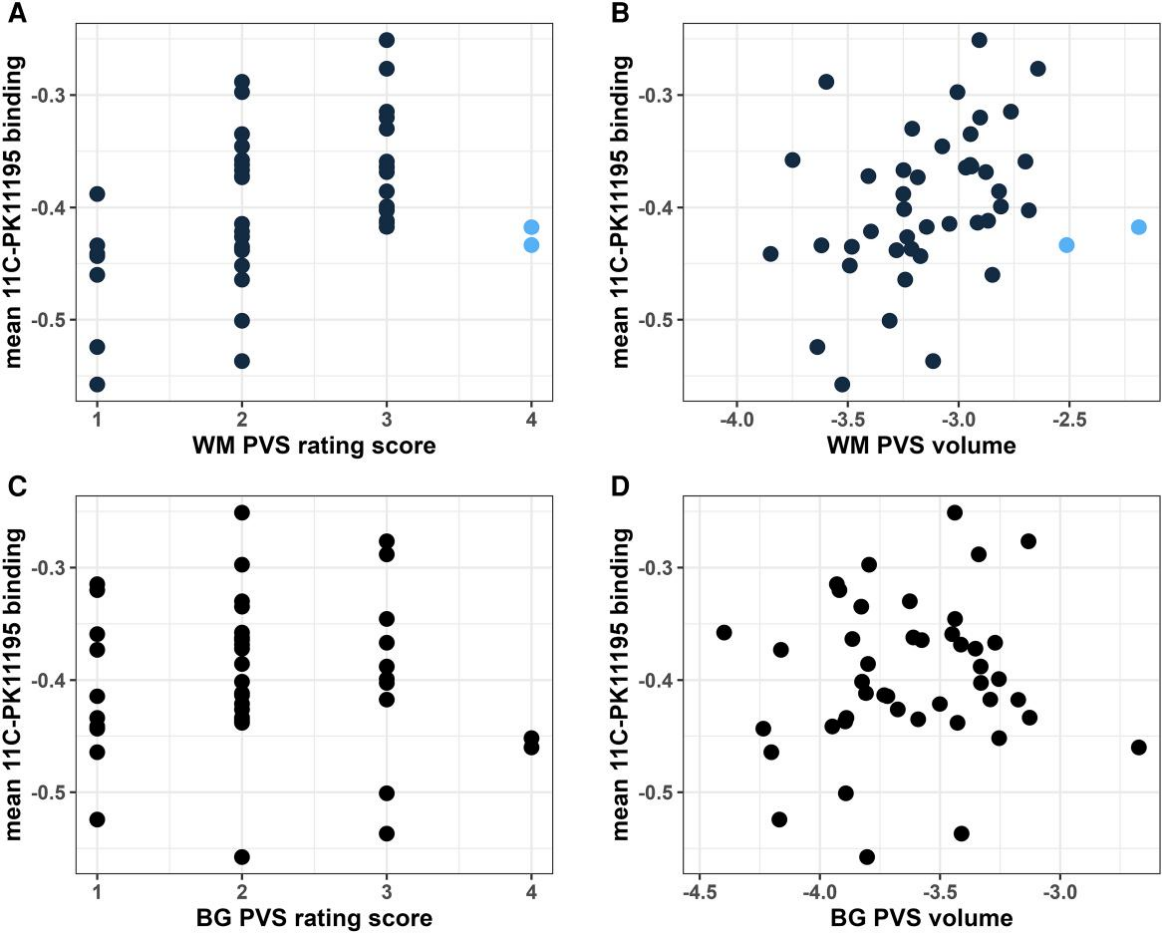
**Point by point mapping of the relationship between perivascular space burden and mean ^11^C-PK11195 binding.** (**A**) Point by point mapping of the correlation between white matter perivascular space (WM PVS) visual rating score and mean ^11^C-PK11195 binding. (**B**) Point by point mapping of the correlation between WM PVS volume and mean ^11^C-PK11195 binding. (**C**) Point by point mapping of the correlation between basal ganglia (BG) PVS visual rating score and mean ^11^C-PK11195 binding. (**D**) Point by point mapping of the correlation between BG PVS volume and mean ^11^C-PK11195 binding. PVS volumes were normalized by brain volume and log-transformed. Blue filled-circles: in two cases, subjects had very high PVS volume but low ^11^C-PK11195 binding.

### Association of PVS markers with ^11^C-PK11195 PET

The relationship between PVS severity, measured by the visual rating and by quantitative volume measurements, with mean BP_ND_ is shown in Fig. 4. Mean BP_ND_ was positively associated with WM PVS assessed on the visual rating (*ρ* = 0.469, FDR-*P* = 0.009). It was also associated with WM PVS volume, but this was no longer significant after FDR correction (β = 0.318, *P* = 0.042, FDR-*P* = 0.076). On the scatterplots, it can be seen that although there was a general increase in mean BP_ND_ in the WM as PVS severity increased, there were a minority of subjects with extremely high WM PVS burden who had relatively low microglial activation (indicated in blue on the scatterplots), suggesting that the relationship was heterogeneous and not uniform across all subjects. In contrast, there was no correlation between BG PVS measured on the rating scale or quantitively with mean BP_ND_ (see Table 2 and Fig. 4).

**Table 2:** The association between ^11^C-PK11195 binding and perivascular space markers

Marker	Mean ^11^C-PK11195 binding		
	Coefficient	*P*-value	FDR-corrected *P*-value
WM PVS visual score	0.469	0.002	**0**.**009**^a^
BG PVS visual score	−0.063	0.694	0.694
Whole PVS volume	0.313	0.045	0.076
WM PVS volume	0.318	0.042	0.076
BG PVS volume	0.139	0.361	0.452

All analyses were adjusted for age and sex. Spearman correlation analysis was used for PVS visual score. Linear regression analysis was used for PVS volume. BG = basal ganglia; FDR = false discovery rate; PVS = perivascular space; WM = white matter.

^a^ *P* < 0.05 after FDR correction.

### Association of PVS markers with BBB permeability

BBB permeability was not associated with any measure of PVS, regardless of region (WM/BG) or method (rating scale/volume) (for detailed results, see Table 3).

**Table 3:** The association between blood–brain barrier permeability and perivascular space markers

Marker	Mean blood–brain barrier permeability		
	Coefficient	*P*-value	FDR-corrected *P*-value
WM PVS visual score	−0.118	0.456	0.456
BG PVS visual score	−0.314	0.043	0.158
Whole PVS volume	−0.254	0.110	0.158
WM PVS volume	−0.242	0.127	0.158
BG PVS volume	−0.239	0.109	0.158

All analyses were adjusted for age and sex. Spearman correlation analysis was used for perivascular space (PVS) visual score. Linear regression analysis was used for PVS volume. BG = basal ganglia; FDR = false discovery rate; WM = white matter.

### Association between blood biomarkers and PVS markers

There was no correlation between PVS markers and 93 blood biomarkers, after adjustment using the FDR for multiple comparisons. Results for individual biomarkers are shown in Supplementary Tables 1 and 2. Likewise, no association was found between PVS markers and any PCs of blood biomarkers after FDR correction (Supplementary Table 3).

### Association between PVS markers and BP_ND_, *K*_i_ and blood biomarkers within the same model

We found that the results were largely consistent with the univariate analysis, with two notable exceptions: the WM PVS visual rating score and mean ^11^C-PK11195 binding (β = 15.682, FDR-*P* = 0.070) and PC3 of blood biomarkers (β = −0.492, FDR-*P* = 0.041). Detailed results are shown in Supplementary Table 4.

## Discussion

In this study, we found the evidence that increased ^11^C-PK11195 binding, but not systemic inflammation, was associated with enlarged PVS. PVS within the white matter were associated with a local increase in ^11^C-PK11195 binding, suggesting microglial activation. Furthermore, within the white matter the overall PVS severity was positively correlated with ^11^C-PK11195 binding. Our data would suggest that PVS in the white matter are associated with inflammation. In contrast, we found no association of BG PVS with ^11^C-PK11195 binding or with increased BBB permeability, either globally or locally within the vicinity of the PVS.

Neuroinflammation has been hypothesized to play a role in glymphatic dysfunction and enlargement of PVS in neurodegenerative diseases and in cSVD,^3,37^ although there has been limited direct evidence supporting this association in cSVD. Our finding of higher ^11^C-PK11195 binding associated with higher WM PVS burden is consistent with one previous study using ^11^C-PK11195, albeit to look for associations with magnetic resonance markers of cSVD within an Alzheimer’s disease and MCI population.^17^ As in our study, that study found an association within the white matter but not in the basal ganglia. The PET values for all tissues were below zero, which be attributable to the reference tissue model used in our study. Although reference tissue methods are well validated and used in the literature,^38-40^ there are limitations associated with them. It is assumed that there is little or no specific binding in the reference population and that the vascular dynamics are similar in the populations. It is possible vascular dynamics could be altered in subjects with cSVD, which might be reflected in reduced apparent binding. However, the relative values within the subjects would be unaffected.

We extended the analysis to determine whether ^11^C-PK11195 binding was increased within the vicinity of enlarged PVS by measuring ligand binding in shells around the PVS and showed increased binding closer to the PVS. This is consistent with a local inflammatory response around the PVS. A similar finding has been reported in multiple sclerosis, in which focal neuroinflammation lesions were accompanied by regional PVS enlargement.^41^ Enlarged PVS are thought to result from multiple factors, including decreased driving force for fluid movement, increased accumulation of waste substances within PVS, and disruption of the aquaporin-4 channel organization.^42^ Microglial activation might play a role in this process by contributing to vascular remodelling,^43^ reducing vessel wall pulsation for fluid within PVS, altering the expression of the aquaporin-4 channel,^44^ and inducing the accumulation of additional waste substances within PVS,^45^ ultimately contributing to PVS enlargement. Alternatively, the PVS enlargement could be a compensatory way to help to alleviate neuroinflammation in the brain.^46^

Increased ^11^C-PK11195 binding around enlarged PVS could also be attributed to the presence of PVS cuffs.^14^ Enlarged PVS typically coincide with the occurrence of PVS cuffs, indicating the formation of leucocyte conglomerates within the PVS before immune cells infiltrate the parenchyma. Consequently, the presence of immune cells from the PVS might stimulate heightened microglial activation around the PVS. However, it is worth noting that perivascular cuffs are commonly observed surrounding postcapillary venules,^47^ whereas MRI-visible enlarged PVS are typically surrounding arterioles.^48^ Nevertheless, two recently published studies in multiple sclerosis and amyotrophic lateral sclerosis suggested that fibrinogen clustered in enlarged PVS were related to microglial activation in the brain.^49,50^

In contrast to the association between PVS and ^11^C-PK11195 binding in the white matter, there was no association in the basal ganglia. Basal ganglia PVS surround the lenticulostriate arteries, known for their higher haemodynamic variability compared with leptomeningeal perforating arteries within white matter PVS.^51,52^ The enlargement of BG PVS might result primarily from direct haemodynamic alterations in lenticulostriate arteries rather than inflammation-related changes.^53,54^

Although ^11^C-PK11195 binding was associated with WM PVS volume overall, there were some patients with extremely high PVS burden and low microglial activation, suggesting some heterogeneity in the relationship. This might be because the pathogenesis of PVS itself is heterogeneous, with inflammation not playing a role in all cases. Alternatively, it might be attributable to a changing role of inflammation across the disease course. For example, in end-stage disease with a very high PVS burden, many of the PVS might be in a more chronic phase, and the inflammatory response could have reduced. Longitudinal studies would be needed to test this.

We did not find any association between PVS markers and BBB permeability. This is consistent with one previous study,^26^ which focused on chronic lacunar stroke patients and found no correlation between PVS burden and gadolinium signal enhancement in the white matter. Both the BBB and the PVS are integral components of the neurovascular unit.^18^ One animal study found that mice with a deficiency of pericytes, an important cell for the BBB, demonstrated enlarged PVS.^55^ Another study observed an augmentation in the perivascular space following the disruption of the BBB induced by transcranial focused ultrasound.^56^ However, the dynamic fluctuations in BBB permeability,^57^ contrasted with the relatively stable and chronic nature of the PVS dysfunction,^58^ might elucidate the lack of association between these two phenomena. Importantly, we validated the DCE-MRI methods for measuring BBB permeability against the CSF-to-serum albumin ratio.

In contrast to the association between ^11^C-PK11195 binding and WM PVS, we found no association between blood biomarkers and all PVS markers after controlling for multiple comparisons for 93 markers, or in a PC analysis. Specifically, we found no association between markers of systemic inflammation, such as C-reactive protein and Interleukin 6 (IL-6 RA), and any PVS markers, consistent with findings from two previous studies.^59,60^ In regional PVS analysis, although we observed a correlation between increased WM PVS burden and previous reported inflammation markers, such as Osteopontin (OPN) and matrix metalloproteinase-3,^61^ before multiple comparison, the results were not significant after FDR correction. This would suggest that systemic inflammation is less important in the pathogenesis of PVS than neuroinflammation, although our ability to detect associations might also have been limited by the moderate sample size for a blood biomarker study.

When analysing the independent associations of mean ^11^C-PK11195 binding, mean BBB permeability, the PCs from blood biomarker analysis with PVS burden in the same model. However, there were two exceptions: the WM PVS visual rating score was not significantly associated with mean ^11^C-PK11195 binding, but it was significantly associated with PC3 of blood biomarkers after adjusting for multiple comparisons. One possible explanation for these differences is the methodological variation between the Spearman correlation analysis and the ‘CLM’ function used in our study. Spearman correlation is a non-parametric test that assesses monotonic relationships between variables without strong assumptions, making it robust to outliers and non-linearity.^62^ In contrast, the CLM function, which treats ordinal variables as dependent, models the log-odds of being in or below a category as a linear function of the predictors, reflecting a different mathematical foundation.^63^ Another factor to consider is the potential interaction between different explanatory variables, which might influence the correlation between PVS burden and these variables in the overall model. Although we did not find any correlation between pairs of explanatory variables in the previous analysis, it is worth noting that the *P*-values for the correlations between mean ^11^C-PK11195 binding and PC3 of blood biomarkers, and between mean BBB permeability and PC2 of blood biomarkers, were both ∼0.100. (Supplementary Table 5) This suggests a possible interaction effect that might not have reached statistical significance, potentially owing to the limited number of participants, which reduced the statistical power of the study.

Our study has a number of strengths. To our knowledge, this is the first study combining blood biomarkers, DCE-MRI and PET-based microglial activation to investigate the association of PVS markers with BBB permeability and neuroinflammation in cSVD. We included a spatial analysis to determine whether PVS were associated with locally increased inflammation.

However, it does also have limitations. Although we have shown an association of ^11^C-PK11195 binding with enlarged PVS, the interpretation of this is complicated by a number of factors. Firstly, the signal seen is negative, suggesting lower uptake than the reference tissue, which suggests a lower ‘inflammation’ in these subjects. This, in turn, suggests that other sources of binding might be influential in the signal seen. TSPO expression has also been linked to processes such as mitochondrial respiration and cellular energy production,^64,65^ which could affect PK11195 binding. It is also possible that reduced blood flow to the brain seen in the subjects with compared with the reference control population might result in the lower than expected binding values seen. Secondly, although ^11^C-PK11195 is widely used as a marker of microglial activation, it might be influenced by off-target and non-specific tissue binding. To address this, we controlled for vascular endothelial binding in the analysis. Thirdly, a recent transcriptomic study suggested that TSPO relates to microglial concentration rather than phenotype. We are unable to differentiate between the pro-inflammatory and anti-inflammatory microglial phenotypes, hence we cannot definitively attribute the changes in binding signal seen to a neuroinflammatory process, and it is possible that the ^11^C-PK11195 signal represents a combination of pro- and anti-inflammatory cells that might not be deleterious when considered together.

## Conclusion

In conclusion, our findings suggest that within the white matter, PVS are associated with microglial activation. Further longitudinal and intervention studies are required to determine whether neuroinflammation plays a causal role in PVS enlargement and glymphatic failure.

## Supplementary Material

awae357_Supplementary_Data

## Data Availability

Deidentified participant data will be made available to interested researchers after approval of a proposal, with a signed data access agreement.
