# Supplementary material for: Perivascular space dysfunction in cerebral small vessel disease is related to neuroinflammation
Source: Brain. 2024 Nov 7;148(5):1540–50. doi: 10.1093/brain/awae357 (PMC12073995; doi:10.1093/brain/awae357)
Supplement: awae357_Supplementary_Data [file awae357_supplementary_data.pdf]

Supplementary Material

Supplementary Fig. 1. The imaging processing workflow for BBB and PET

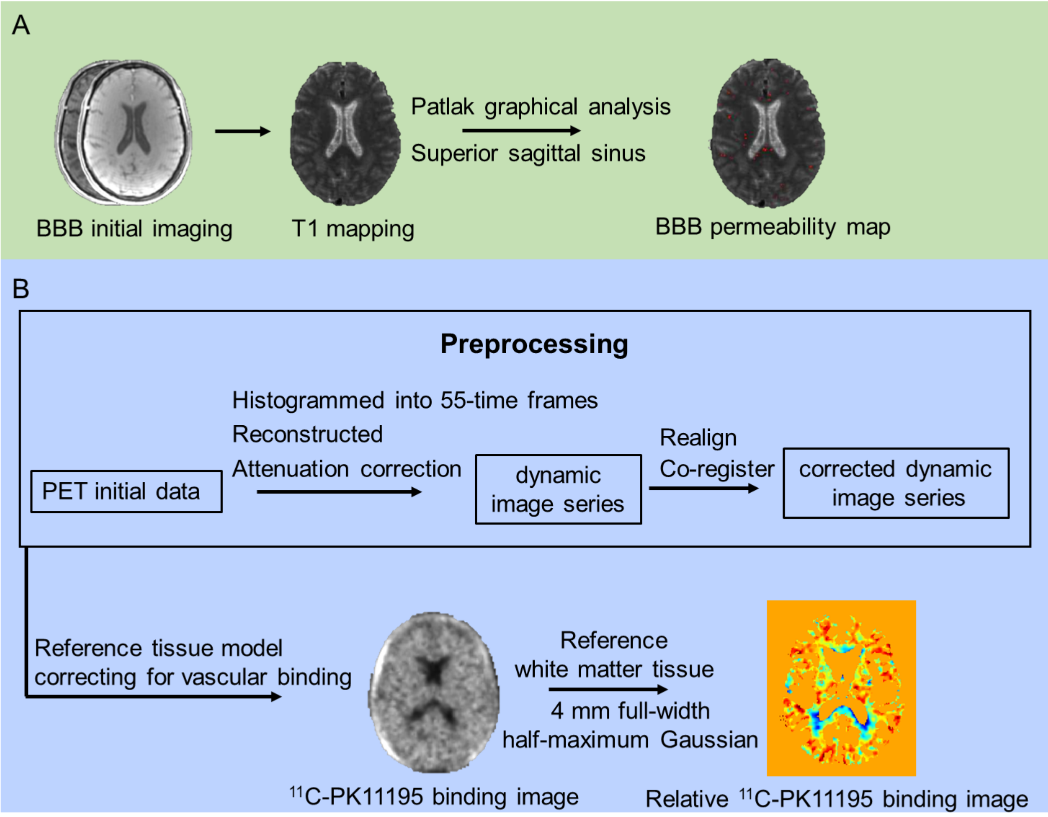

A. BBB processing workflow

B. PET processing workflow

Supplementary Fig. 2. Principle analysis for the blood biomarkers in cSVD patients

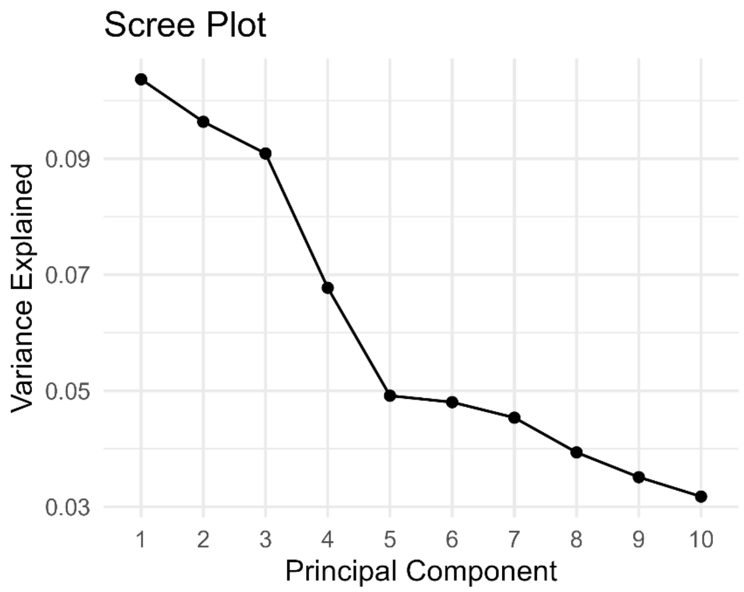

Supplementary Table1 The association between blood biomarker and PVS burden in different regions

Blood biomarker	WM PVS rating score			BG PVS rating score			WM PVS volume			BG PVS volume		
	rho	p	FDR-p	rho	p	FDR-p	r	p	FDR-p	r	p	FDR-p
CRP	-0.129	0.403	0.930	0.125	0.419	0.930	-0.271	0.075	0.838	0.081	0.600	0.956
TNFRSF14	-0.042	0.786	0.973	-0.071	0.647	0.973	-0.091	0.557	0.949	-0.033	0.832	0.973
LDL.receptor	-0.103	0.506	0.949	0.057	0.715	0.973	0.017	0.911	0.989	0.056	0.720	0.973
ITGB2	0.081	0.602	0.956	-0.015	0.925	0.990	0.074	0.632	0.969	0.209	0.174	0.893
IL.17RA	0.078	0.614	0.966	0.045	0.773	0.973	-0.028	0.857	0.973	0.077	0.621	0.966
TNF.R2	0.063	0.686	0.973	0.001	0.996	1.000	0.194	0.206	0.924	-0.000	1.000	1.000
MMP.9	0.003	0.985	0.996	0.151	0.329	0.927	0.186	0.226	0.924	0.128	0.408	0.930
EPHB4	-0.008	0.959	0.996	0.172	0.264	0.927	0.073	0.637	0.969	0.125	0.420	0.930
IL2.RA	0.004	0.977	0.996	-0.094	0.543	0.949	0.078	0.615	0.966	0.107	0.490	0.949
OPG	-0.027	0.863	0.973	-0.144	0.351	0.927	0.030	0.849	0.973	0.148	0.339	0.927
ALCAM	-0.077	0.618	0.966	0.015	0.924	0.990	0.180	0.241	0.924	0.153	0.322	0.927
TFF3	-0.160	0.298	0.927	0.136	0.379	0.927	0.105	0.497	0.949	0.255	0.095	0.862
SELP	0.034	0.827	0.973	0.222	0.148	0.893	0.054	0.727	0.973	0.161	0.297	0.927
CSTB	-0.195	0.206	0.924	0.055	0.723	0.973	-0.219	0.153	0.893	0.126	0.414	0.930
MCP.1	0.047	0.760	0.973	0.169	0.272	0.927	0.033	0.831	0.973	0.151	0.327	0.927
CD163	-0.048	0.758	0.973	0.204	0.183	0.893	-0.191	0.215	0.924	0.075	0.630	0.969
Gal.3	-0.039	0.803	0.973	-0.151	0.327	0.927	-0.140	0.366	0.927	-0.086	0.577	0.956
GRN	0.155	0.315	0.927	0.019	0.903	0.988	0.139	0.369	0.927	-0.023	0.883	0.987
NT.proBNP	-0.092	0.552	0.949	-0.089	0.566	0.955	0.068	0.659	0.973	0.090	0.561	0.949
BLM.hydrolase	0.019	0.902	0.988	-0.244	0.110	0.862	0.034	0.827	0.973	-0.067	0.668	0.973
PLC	0.362	0.016	0.629	-0.130	0.400	0.930	0.314	0.038	0.751	-0.184	0.233	0.924
LTBR	0.019	0.905	0.988	-0.087	0.576	0.956	0.006	0.971	0.996	-0.102	0.510	0.949

Notch.3	-0.003	0.982	0.996	-0.324	0.032	0.751	0.136	0.379	0.927	0.050	0.746	0.973
TIMP4	0.030	0.849	0.973	-0.149	0.333	0.927	-0.020	0.896	0.988	0.146	0.345	0.927
CNTN1	0.157	0.310	0.927	-0.217	0.158	0.893	0.068	0.661	0.973	-0.126	0.415	0.930
CDH5	0.161	0.297	0.927	-0.061	0.692	0.973	-0.021	0.895	0.988	-0.128	0.407	0.930
TLT.2	-0.182	0.238	0.924	-0.057	0.715	0.973	-0.140	0.363	0.927	-0.099	0.524	0.949
FABP4	-0.112	0.469	0.949	0.102	0.511	0.949	-0.163	0.289	0.927	0.174	0.259	0.927
TFPI	0.044	0.778	0.973	0.348	0.020	0.744	0.043	0.780	0.973	0.207	0.177	0.893
PAI	0.123	0.426	0.930	0.433	0.003	0.629	0.093	0.548	0.949	0.224	0.143	0.893
CCL24	0.287	0.059	0.838	0.239	0.119	0.886	0.244	0.110	0.862	0.004	0.980	0.996
TR	-0.148	0.337	0.927	0.100	0.519	0.949	-0.149	0.335	0.927	0.178	0.247	0.924
TNFRSF10C	-0.293	0.054	0.838	0.128	0.409	0.930	-0.318	0.036	0.751	0.100	0.519	0.949
GDF.15	-0.099	0.523	0.949	0.118	0.446	0.936	-0.161	0.297	0.927	0.097	0.533	0.949
SELE	0.070	0.649	0.973	0.247	0.107	0.862	0.120	0.438	0.936	0.034	0.824	0.973
AZU1	0.148	0.337	0.927	-0.015	0.924	0.990	0.297	0.050	0.838	-0.084	0.588	0.956
DLK.1	0.081	0.600	0.956	-0.167	0.279	0.927	0.189	0.220	0.924	-0.216	0.160	0.893
SPON1	-0.262	0.085	0.851	0.011	0.944	0.993	-0.050	0.747	0.973	-0.000	0.999	1.000
MPO	-0.217	0.158	0.893	-0.009	0.952	0.995	-0.065	0.677	0.973	-0.014	0.927	0.990
CXCL16	-0.052	0.737	0.973	0.018	0.907	0.989	-0.083	0.591	0.956	-0.019	0.903	0.988
IL.6RA	0.143	0.354	0.927	-0.050	0.747	0.973	0.029	0.850	0.973	-0.049	0.750	0.973
RETN	0.269	0.078	0.838	0.106	0.492	0.949	0.117	0.449	0.936	0.033	0.833	0.973
IGFBP.1	0.137	0.376	0.927	-0.133	0.389	0.930	0.153	0.321	0.927	-0.076	0.624	0.966
CHIT1	-0.063	0.683	0.973	-0.204	0.184	0.893	0.060	0.701	0.973	-0.098	0.529	0.949
TR.AP	0.019	0.903	0.988	-0.335	0.026	0.751	-0.052	0.736	0.973	-0.101	0.514	0.949
GP6	-0.048	0.755	0.973	-0.179	0.246	0.924	-0.044	0.777	0.973	-0.132	0.395	0.930
PSP.D	0.007	0.965	0.996	-0.266	0.081	0.838	-0.102	0.508	0.949	-0.109	0.481	0.949
PI3	0.060	0.698	0.973	-0.499	0.001	0.318	0.088	0.568	0.955	-0.266	0.081	0.838

Ep.CAM	0.205	0.183	0.893	-0.192	0.212	0.924	0.051	0.743	0.973	-0.136	0.378	0.927
AP.N	0.251	0.101	0.862	-0.046	0.764	0.973	0.179	0.245	0.924	-0.145	0.346	0.927
AXL	0.150	0.330	0.927	0.171	0.266	0.927	0.263	0.085	0.851	-0.043	0.783	0.973
IL.1RT1	0.047	0.762	0.973	-0.182	0.238	0.924	0.113	0.465	0.943	-0.096	0.535	0.949
MMP.2	-0.110	0.478	0.949	-0.227	0.139	0.893	-0.004	0.981	0.996	-0.096	0.535	0.949
FAS	-0.063	0.685	0.973	-0.167	0.278	0.927	-0.095	0.542	0.949	0.010	0.948	0.993
MB	-0.039	0.802	0.973	-0.103	0.504	0.949	-0.011	0.945	0.993	0.066	0.671	0.973
TNFSF13B	0.041	0.791	0.973	-0.152	0.324	0.927	-0.004	0.980	0.996	-0.083	0.591	0.956
PRTN3	0.138	0.372	0.927	-0.004	0.979	0.996	0.056	0.720	0.973	0.115	0.459	0.942
PCSK9	0.056	0.720	0.973	0.065	0.674	0.973	-0.066	0.669	0.973	0.205	0.182	0.893
U.PAR	0.203	0.186	0.896	0.235	0.125	0.886	0.171	0.268	0.927	0.151	0.327	0.927
OPN	0.382	0.010	0.629	0.204	0.184	0.893	0.420	0.004	0.629	0.063	0.686	0.973
CTSD	0.412	0.005	0.629	0.117	0.449	0.936	0.346	0.021	0.744	0.015	0.922	0.990
PGLYRP1	-0.034	0.826	0.973	-0.150	0.330	0.927	0.063	0.686	0.973	-0.027	0.860	0.973
CPA1	-0.084	0.589	0.956	-0.138	0.373	0.927	-0.124	0.421	0.930	-0.010	0.947	0.993
JAM.A	-0.113	0.464	0.943	-0.226	0.140	0.893	-0.107	0.488	0.949	-0.097	0.530	0.949
Gal.4	-0.368	0.014	0.629	0.197	0.201	0.924	-0.331	0.028	0.751	0.217	0.157	0.893
IL.1RT2	-0.218	0.155	0.893	0.171	0.267	0.927	0.014	0.930	0.990	0.252	0.099	0.862
SHPS.1	0.098	0.525	0.949	-0.084	0.590	0.956	0.076	0.625	0.966	-0.117	0.448	0.936
CCL15	0.028	0.855	0.973	-0.230	0.134	0.893	-0.011	0.945	0.993	0.030	0.847	0.973
CASP.3	-0.073	0.639	0.969	0.212	0.166	0.893	-0.122	0.430	0.933	0.152	0.325	0.927
uPA	0.055	0.722	0.973	0.147	0.340	0.927	-0.099	0.523	0.949	0.021	0.893	0.988
CPB1	0.226	0.140	0.893	0.291	0.056	0.838	0.037	0.809	0.973	-0.140	0.363	0.927
CHI3L1	0.024	0.875	0.980	0.266	0.081	0.838	-0.115	0.459	0.942	0.019	0.904	0.988
ST2	-0.014	0.928	0.990	0.098	0.525	0.949	0.004	0.980	0.996	-0.092	0.551	0.949
t.PA	-0.047	0.762	0.973	0.027	0.862	0.973	0.158	0.304	0.927	-0.005	0.975	0.996

SCGB3A2	-0.036	0.815	0.973	-0.093	0.548	0.949	0.066	0.670	0.973	-0.015	0.925	0.990
EGFR	-0.208	0.175	0.893	-0.209	0.173	0.893	-0.128	0.406	0.930	-0.033	0.831	0.973
IGFBP.7	-0.160	0.300	0.927	0.234	0.126	0.886	0.043	0.783	0.973	0.380	0.011	0.629
CD93	-0.215	0.161	0.893	0.284	0.062	0.838	-0.057	0.712	0.973	0.233	0.129	0.886
IL.18BP	-0.278	0.068	0.838	0.318	0.036	0.751	-0.246	0.108	0.862	0.180	0.242	0.924
COL1A1	-0.280	0.066	0.838	0.332	0.028	0.751	-0.201	0.191	0.909	0.273	0.073	0.838
PON3	-0.205	0.182	0.893	0.042	0.784	0.973	-0.184	0.233	0.924	0.145	0.348	0.927
CTSZ	0.056	0.719	0.973	0.152	0.324	0.927	0.234	0.126	0.886	0.178	0.246	0.924
MMP.3	0.166	0.280	0.927	0.182	0.238	0.924	0.368	0.014	0.629	0.177	0.249	0.924
RARRES2	0.110	0.476	0.949	0.257	0.092	0.862	0.130	0.402	0.930	-0.082	0.596	0.956
ICAM.2	0.406	0.006	0.629	0.323	0.032	0.751	0.328	0.029	0.751	0.050	0.747	0.973
KLK6	0.148	0.338	0.927	0.083	0.592	0.956	0.081	0.603	0.956	-0.069	0.658	0.973
PDGF.subunit.A	-0.039	0.801	0.973	0.159	0.303	0.927	-0.073	0.637	0.969	0.135	0.384	0.927
TNF.R1	-0.016	0.919	0.990	0.208	0.176	0.893	-0.047	0.763	0.973	0.037	0.812	0.973
IGFBP.2	-0.222	0.147	0.893	-0.038	0.808	0.973	-0.248	0.104	0.862	-0.276	0.070	0.838
vWF	-0.285	0.061	0.838	-0.130	0.402	0.930	-0.191	0.213	0.924	-0.250	0.101	0.862
PECAM.1	-0.130	0.400	0.930	-0.050	0.748	0.973	-0.035	0.821	0.973	-0.315	0.038	0.751
MEPE	-0.288	0.058	0.838	0.148	0.337	0.927	-0.260	0.089	0.852	0.153	0.320	0.927
CCL16	-0.143	0.356	0.927	0.251	0.101	0.862	-0.138	0.370	0.927	0.312	0.039	0.751

Supplementary Table 2 The association between blood biomarker and whole brain PVS volume

Blood biomarker	Whole PVS volume		
	r	p	FDR-p
CRP	-0.177	0.250	0.924
TNFRSF14	-0.140	0.366	0.927
LDL.receptor	0.054	0.729	0.973
ITGB2	0.200	0.194	0.916
IL.17RA	-0.028	0.856	0.973
TNF.R2	0.180	0.243	0.924
MMP.9	0.233	0.128	0.886
EPHB4	0.102	0.509	0.949
IL2.RA	0.127	0.410	0.930
OPG	0.090	0.560	0.949
ALCAM	0.237	0.121	0.886
TFF3	0.171	0.267	0.927
SELP	0.097	0.532	0.949
CSTB	-0.128	0.406	0.930
MCP.1	0.081	0.601	0.956
CD163	-0.154	0.320	0.927
Gal.3	-0.122	0.432	0.934
GRN	0.119	0.442	0.936
NT.proBNP	0.081	0.600	0.956
BLM.hydrolase	0.034	0.825	0.973
PLC	0.211	0.169	0.893
LTBR	-0.042	0.784	0.973
Notch.3	0.136	0.379	0.927
TIMP4	0.067	0.668	0.973
CNTN1	0.003	0.987	0.996
CDH5	-0.084	0.588	0.956
TLT.2	-0.172	0.263	0.927
FABP4	-0.094	0.544	0.949
TFPI	0.096	0.537	0.949
PAI	0.124	0.422	0.930
CCL24	0.180	0.242	0.924
TR	-0.068	0.663	0.973
TNFRSF10C	-0.223	0.146	0.893
GDF.15	-0.107	0.490	0.949
SELE	0.097	0.533	0.949
AZU1	0.213	0.166	0.893
DLK.1	0.066	0.670	0.973
SPON1	-0.039	0.800	0.973

MPO	-0.045	0.771	0.973
CXCL16	-0.054	0.727	0.973
IL.6RA	0.042	0.784	0.973
RETN	0.120	0.439	0.936
IGFBP.1	0.102	0.509	0.949
CHIT1	0.001	0.993	0.998
TR.AP	-0.074	0.635	0.969
GP6	-0.058	0.707	0.973
PSP.D	-0.098	0.526	0.949
PI3	-0.021	0.894	0.988
Ep.CAM	-0.004	0.981	0.996
AP.N	0.069	0.656	0.973
AXL	0.164	0.289	0.927
IL.1RT1	0.041	0.792	0.973
MMP.2	-0.037	0.811	0.973
FAS	-0.068	0.659	0.973
MB	0.017	0.911	0.989
TNFSF13B	-0.012	0.936	0.993
PRTN3	0.118	0.446	0.936
PCSK9	0.032	0.836	0.973
U.PAR	0.175	0.255	0.927
OPN	0.375	0.012	0.629
CTSD	0.283	0.062	0.838
PGLYRP1	0.032	0.838	0.973
CPA1	-0.132	0.393	0.930
JAM.A	-0.160	0.300	0.927
Gal.4	-0.208	0.175	0.893
IL.1RT2	0.099	0.521	0.949
SHPS.1	0.008	0.961	0.996
CCL15	-0.031	0.843	0.973
CASP.3	-0.047	0.764	0.973
uPA	-0.062	0.690	0.973
CPB1	-0.015	0.923	0.990
CHI3L1	-0.120	0.437	0.936
ST2	-0.068	0.663	0.973
t.PA	0.115	0.457	0.942
SCGB3A2	0.045	0.773	0.973
EGFR	-0.126	0.413	0.930
IGFBP.7	0.187	0.225	0.924
CD93	0.052	0.736	0.973
IL.18BP	-0.143	0.355	0.927
COL1A1	-0.067	0.665	0.973
PON3	-0.115	0.457	0.942
CTSZ	0.266	0.081	0.838
MMP.3	0.392	0.009	0.629

RARRES2	0.086	0.579	0.956
ICAM.2	0.300	0.048	0.838
KLK6	0.012	0.940	0.993
PDGF.subunit.A	-0.011	0.945	0.993
TNF.R1	-0.013	0.932	0.991
IGFBP.2	-0.329	0.029	0.751
vWF	-0.260	0.088	0.852
PECAM.1	-0.139	0.367	0.927
MEPE	-0.157	0.308	0.927
CCL16	-0.020	0.898	0.988

Supplementary Table 3 The association between PVS markers and each principal component of blood biomarkers

	PC1			PC2			PC3		
	Coef	p	FDR-p	Coef	p	FDR-p	Coef	p	FDR-p
WM PVS visual score	0.027	0.864	0.917	0.197	0.201	0.436	-0.315	0.037	0.262
BG PVS visual score	-0.418	0.005	0.072	0.294	0.052	0.262	-0.032	0.837	0.917
Whole PVS volume	-0.037	0.814	0.917	0.131	0.397	0.662	-0.209	0.174	0.436
WM PVS volume	0.016	0.917	0.917	0.184	0.233	0.436	-0.236	0.123	0.436
BG PVS volume	-0.195	0.204	0.436	-0.044	0.779	0.917	0.023	0.883	0.917

All analyses were adjusted for age and sex. Spearman correlation analysis was used for PVS visual score. Pearson correlation analysis was used for PVS volume; PVS, perivascular space; WM, white matter; BG, basal ganglia.

Supplementary Table 4 Multiple linear regression between PVS burden and mean BBB permeability, mean ^{11}C-PK11195 binding, and PCs

	**Whole PVS volume**			**White matter PVS volume**			**Basal ganglia PVS volume**			**White matter visual rating**			**Basal ganglia visual rating**		
**P-value for the model fitting**	0.273			0.506			0.103			**0.002***			**0.008***		
	β	p-value	FDR-p	β	p-value	FDR-p	β	p-value	FDR-p	β	p-value	FDR-p	β	p-value	FDR-p
Age	0.005	0.299	0.374	0.001	0.810	0.810	0.010	0.088	0.220	-0.055	0.180	0.301	0.095	0.015	0.074
Sex	-0.046	0.656	0.942	-0.008	0.942	0.942	-0.166	0.201	0.942	0.075	0.930	0.942	-0.224	0.780	0.942
Mean BBB permeability	-1.448	0.319	0.399	-1.369	0.405	0.405	-2.626	0.150	0.348	-15.629	0.209	0.348	-31.333	0.034*	0.168
Mean ^{11}C-PK11195 binding	1.702	0.042*	0.070	1.484	0.112	0.140	2.319	0.027*	0.070	15.682	0.035*	0.070	5.781	0.365	0.365
PC1	0.006	0.761	0.935	0.002	0.935	0.935	0.003	0.901	0.935	-0.112	0.494	0.935	-0.084	0.571	0.935
PC2	0.005	0.752	0.872	0.011	0.521	0.868	-0.003	0.872	0.872	0.309	0.028*	0.139	0.241	0.069	0.174
PC3	-0.008	0.606	0.758	-0.014	0.446	0.744	0.015	0.440	0.744	-0.492	0.008*	**0.041***	-0.012	0.928	0.928

PVS, perivascular space

Supplementary Table 5 The association between mean BBB permeability, mean ^{11}C-PK11195 binding, and PCs of blood biomarkers

	**PC1**		**PC2**		**PC3**	
	r	p	r	p	r	p
Mean ^{11}C-PK11195 binding	0.217	0.233	0.190	0.298	-0.254	0.161
Mean BBB permeability	0.115	0.531	0.294	0.102	0.121	0.510
